# Quantitative Ultrashort Echo Time (UTE) Magnetic Resonance Imaging of Bone: An Update

**DOI:** 10.3389/fendo.2020.567417

**Published:** 2020-09-18

**Authors:** Ya-Jun Ma, Saeed Jerban, Hyungseok Jang, Douglas Chang, Eric Y. Chang, Jiang Du

**Affiliations:** ^1^Department of Radiology, University of California, San Diego, San Diego, CA, United States; ^2^Department of Orthopedic Surgery, University of California, San Diego, San Diego, CA, United States; ^3^Research Service, Veterans Affairs San Diego Healthcare System, San Diego, CA, United States

**Keywords:** MRI, cortical bone, trabecular bone, UTE, water contents, macromolecular fraction, bone mineral density

## Abstract

Bone possesses a highly complex hierarchical structure comprised of mineral (~45% by volume), organic matrix (~35%) and water (~20%). Water exists in bone in two forms: as bound water (BW), which is bound to bone mineral and organic matrix, or as pore water (PW), which resides in Haversian canals as well as in lacunae and canaliculi. Magnetic resonance (MR) imaging has been increasingly used for assessment of cortical and trabecular bone. However, bone appears as a signal void on conventional MR sequences because of its short T2^*^. Ultrashort echo time (UTE) sequences with echo times (TEs) 100–1,000 times shorter than those of conventional sequences allow direct imaging of BW and PW in bone. A series of quantitative UTE MRI techniques has been developed for bone evaluation. UTE and adiabatic inversion recovery prepared UTE (IR-UTE) sequences have been developed to quantify BW and PW. UTE magnetization transfer (UTE-MT) sequences have been developed to quantify collagen backbone protons, and UTE quantitative susceptibility mapping (UTE-QSM) sequences have been developed to assess bone mineral.

## Background

Osteoporosis (OP) is a metabolic bone disease which affects more than 10 million people in the United States and leads to over two million fractures every year (1). For many patients, OP can result in long-term disability and death. Approximately 80% of the skeletal mass is composed of cortical bone, the bone layer where most fractures in old age occur (2). However, OP always progresses in tandem with large trabecular bone deterioration (3). It is crucial then to understand the underlying constituent components of cortical and trabecular bone and their fractions more thoroughly by elucidating the mechanical and functional relationship between the ways they degenerate and fail. The development of non-invasive imaging techniques to evaluate bone constituent components, their stability, and functionality is a critical and driving force in these explorations.

Bone has a highly complex hierarchical structure (4) comprised of mineral (>40% by volume), organic matrix (>30%) and water (~20%) at cortical sites (5, 6). Bone mineral provides stiffness and strength, particularly at compression loading, while collagen provides ductility and the crucial ability to absorb energy before fracture. Water exists in cortical and trabecular bone at multiple locations and in various states (5, 6): in trabecular bone, water exists primarily in combination with fat in bone marrow, typically occupying over 80% of bone volume, but sometimes occupying over 95% of bone volume in OP (3, 7). In normal cortical bone, a large portion of water is bound to either crystals of apatite-like bone mineral or to the organic matrix (8–14). A smaller fraction of this water exists in “free” form and resides in pores, including Haversian canals (10–200 μm), lacunae (1–10 μm) and canaliculi (0.1–1 μm) (5, 8). Bound water (BW) and pore water (PW) generally contribute differently to the mechanical properties of bone (15, 16). BW is directly related to bone strength and toughness, while PW is inversely related to modulus of elasticity. Water, in general, is responsible for bone's viscoelastic properties, such that bone drying (e.g., long periods at room temperature or short periods at higher temperatures) results in a decrease of the bone toughness through reductions in strength and fracture strain (17).

Bone imaging has been performed in clinical evaluations since Roentgen introduced the first radiograph in 1895. Standard evaluation of bone in clinics has been focused on measuring bone mineral density (BMD) using x-ray-based techniques such as dual-energy X-ray absorptiometry (DEXA) and quantitative computed tomography (QCT). The organic matrix, water and fat, which together represent ~55% and ~80% of cortical and trabecular bone by volume, respectively, only make minor contributions to the signal obtained by the standard x-ray-based techniques currently available in clinical settings (17–20). Measurement of BMD alone is only able to predict fractures with a success rate of 30–50% (21–23). While overall fracture risk increases 13-fold from age 60 to 80, it is estimated that the observed decrease in BMD during this period can only account for a doubling of this fracture risk (24). A major absent factor in bone fracture risk estimation is the contribution of bone organic matrix and water to the overarching biomechanical properties of bone.

Conventional magnetic resonance imaging (MRI) provides a non-invasive assessment of protons in soft tissues and avoids the potential harm associated with x-ray-based imaging techniques. However, cortical bone has a short apparent transverse relaxation time (T2^*^), rendering it invisible when studied using conventional clinical MRI pulse sequences with echo times (TEs) of a few milliseconds or longer (25, 26). The lack of direct signal obtained from bone makes it impossible to quantify the MR relaxation times (e.g., T1 and T2^*^), magnetization transfer ratio (MTR) and volume concentration of various bone compartments. To address this shortcoming and take advantage of both MRI's safety profile and its excellent assessment of soft tissues such as tendon (27) and muscle, a benefit not available in x-ray-based techniques, a number of advanced MRI techniques have recently been developed to evaluate bone more effectively (14, 28–30). Among recently developed MRI techniques, ultrashort echo time (UTE) sequences have emerged as a technique capable of directly imaging cortical bone and providing a number of quantitative measurements (14, 28–30). The wide range of bone quantifications available using UTE MRI and several reported validation investigations have led the field of quantitative MRI imaging to gravitate toward UTE MRI technique. In addition to UTE MRI technique (focus of this review), Zero echo time (ZTE) sequence, which utilizes a short rectangular excitation pulse during the fully ramped up readout gradient followed by fast radial sampling (31–34), is an alternative approach for bone imaging. Furthermore, sweep imaging with Fourier transformation (SWIFT), a frequency-modulated pulse sequence with interleaved transmit-receive operation (35, 36), is another method that has been used for bone imaging. ZTE and SWIFT are silent MR sequences and more sensitive in detecting MR signal of the very shortest T2^*^ component of bone in comparison with UTE sequence. However, UTE sequence is more flexible in that it allows for adjustment of echo time and flip angle. Therefore, more biomarkers, such as pore water fraction, can be obtained using UTE techniques for bone quantification. Using a higher flip angle, UTE sequences can achieve higher image SNR, as well.

The following discussion describes UTE MRI techniques which have been developed for quantitative imaging of cortical and trabecular bone in order to estimate different components of bone and predict its microstructural and mechanical properties. A summary of the reviewed techniques and their applications is presented in Table 1.

**Table 1:** Comparing quantitative MRI techniques for bone imaging.

	**UTE MRI technique**	**Quantification**	**Scan time-efficiency**	**Predicted bone characteristics**
**Cortical bone**	Basic UTE (+ phantom imaging) (37–45)	Total water proton density	High	- Significantly correlated positively with cortical bone porosity and negatively with BMD (μCT) (44, 45)
	IR-UTE (+ phantom imaging) (41–44, 46)	Bound water proton density	Moderate	- Significantly correlated positively with cortical bone stiffness, strength, and toughness to fracture (47, 48)
	DAEF-UTE (+ phantom imaging) (41, 47)	Pore water proton density	Moderate	- Significantly correlated positively with bone porosity (μCT) and negatively with stiffness, strength, and toughness to fracture (47, 48)
	IR-UTE and UTE subtraction (+ phantom imaging) (38, 44, 49)	Pore water proton density	Moderate	- Significantly correlated positively with cortical bone porosity and negatively with BMD (μCT) (44)
	Bicomponent UTE fitting (12, 16, 50–52)	T2*s of bound and pore water, as well as bound water to total water ratio	Low	- Pore water fraction was significantly correlated positively with cortical bone porosity (μCT and histomorphometry) and negatively with BMD, stiffness, and strength (negatively) (16, 50, 51, 53). Correlations of bound water fraction were inverse.
	Tricomponent UTE fitting (53, 54)	T2*s of bound, pore water and fat, as well as bound and fat to total water ratios	Low	- Pore water fraction was significantly correlated positively with cortical bone porosity (μCT) and negatively with BMD, stiffness, and strength (53, 54). Correlations of bound water fraction were inverse.
	UTE to IR-UTE signal fraction (40)	Total and bound water ratio	Moderate	- Significantly correlated positively with cortical bone porosity (μCT) and age (40).
	Dual TE signal fraction (55, 56)	Pore and total water ratio	High	- Significantly correlated positively with cortical bone porosity (μCT) and donor age and negatively with mechanical stiffness and collagen estimation from near infrared spectroscopy (55, 56).
	Basic UTE signal decomposition model (57)	Bound and pore water ratio	High	- Pore water fraction was significantly correlated positively with subject age (57). Correlations of bound water fraction were inverse.
	UTE-MT modeling (44, 51, 58–62)	Macromolecular proton to total proton ratio	Low	- Significantly correlated negatively with cortical bone porosity (μCT and Histomorphometry) and positively with BMD, stiffness, and strength (44, 51, 58, 61).
	UTE-MT modeling and Basic UTE (+ phantom imaging) (44)	Macromolecular proton density	Low	- Significantly correlated negatively with cortical bone porosity (μCT) and subject age (44).
	UTE QSM (63, 64)	Magnetic susceptibility (BMD estimation)	Low	- Significantly correlated negatively with cortical bone porosity (μCT) and positively with BMD (64)
	Basic UTE at 31P frequency (42, 43, 65, 66)	Phosphorous content (BMD estimation)	Moderate	- Feasibility studies were performed (66).
**Trabecularbone**	SPIR UTE (67)	Bound water T2*	Moderate	- Correlated positively with cortical bone porosity (μCT) (67)
	IR-UTE (68)	Bound water content	Moderate	- Feasibility studies were performed (68).

## UTE MRI Quantification of Cortical Bone

### UTE Imaging of Total Water (TW) in Cortical Bone

UTE sequences, which are MRI pulse sequences which utilize TEs <100 μs, can visualize both BW and PW (37, 38, 69). While previous UTE technical developments were focused on reducing TE in an effort to continue improving the detection of signal from bone, the most recent UTE technical developments have been centered on improving the selective quantifications of BW, PW, and other bone components, including their relaxation times (e.g., T1 and T2^*^), fractions, and volume concentrations.

Total water (TW) content of cortical bone can be estimated by comparing the UTE MRI signal of bone with that of an external reference with known proton density (37–43), though the resulting estimated content must be corrected for the difference in T2^*^ and T1 values of bone and the external reference (44). Several research groups have used a mixture of distilled water and deuterated water (e.g., 20% H_2_O and 80% D2O, 22 mol/L ^1^H) doped with MnCl2 and titrated to match the effective T2^*^ of cortical bone (e.g., T2^*^ ≈ 0.4 ms) as the external reference for this estimation technique (38, 39, 41, 43, 44), but, notably, any phantom with known apparent proton density and with a range of MRI properties similar to bone, such as a rubber eraser, can be used (45). Significant correlations have been reported between the estimated TW content in human cortical bone and its microstructural properties (44, 45).

For accurate estimation of TW content, we should consider, first, the difference between relaxation times of cortical bone and the external phantom, second, the spatial variation of coil sensitivity in scanned field of view (FOV), and third, the duration of radiofrequency (RF) pulse and its inhomogeneity [or actual flip angle (FA)] (38, 70). Due to the short T1 in cortical bone, the T1 effect on the TW content calculation can be neglected if one uses a relatively low FA combined with a relatively high repetition time (TR) in a proton density (PD)-weighted UTE sequence (45).

Although basic UTE MRI cannot provide high contrast for visualizing bone alone, this fast MRI imaging technique has the potential to provide an initial evaluation of bone microstructure that can facilitate early diagnosis and monitoring of OP in cross-sectional and longitudinal investigations.

### IR-UTE Imaging of BW in Cortical Bone

Most clinical MR scanners can utilize adiabatic inversion recovery-based UTE (IR-UTE) sequences and long T2-saturated UTE sequences to specifically image BW (47, 71), and comparing the IR-UTE signal from cortical bone with that of an external reference can be used to estimate BW content (41–44, 46). BW content quantification based on the IR-UTE sequence requires the assumption that PW signal nulling is efficient (38). With that in mind, PW content in cortical bone can be calculated indirectly by subtracting the IR-UTE-measured BW content from UTE-measured TW content (38, 44, 49).

As described in earlier studies, higher BW T1 (T_1−BW_) values result in BW content underestimation if appropriate T1 compensation is not considered (44). When applying T1 compensation in BW content assessment, BW signal is assumed as an exponential function of T_1−BW_; therefore, if the assumed T_1−BW_ value used for compensation is higher than the true value, this would result in a significant overestimation of BW content (44). While Tan et al. reported using a short T_1−BW_ of 112ms at 3T *in vivo* (72), a value consistent with earlier reports by the same group (44, 73), other studies have reported using a T_1−BW_ equal to 290 ms at 3T (41, 43). BW content estimations have been reported to show significant correlations with mechanical properties of human cortical bone (47). However, some studies were not able to reproduce significant correlations between BW content estimation and cortical bone microstructure (44).

**Figure 1:**
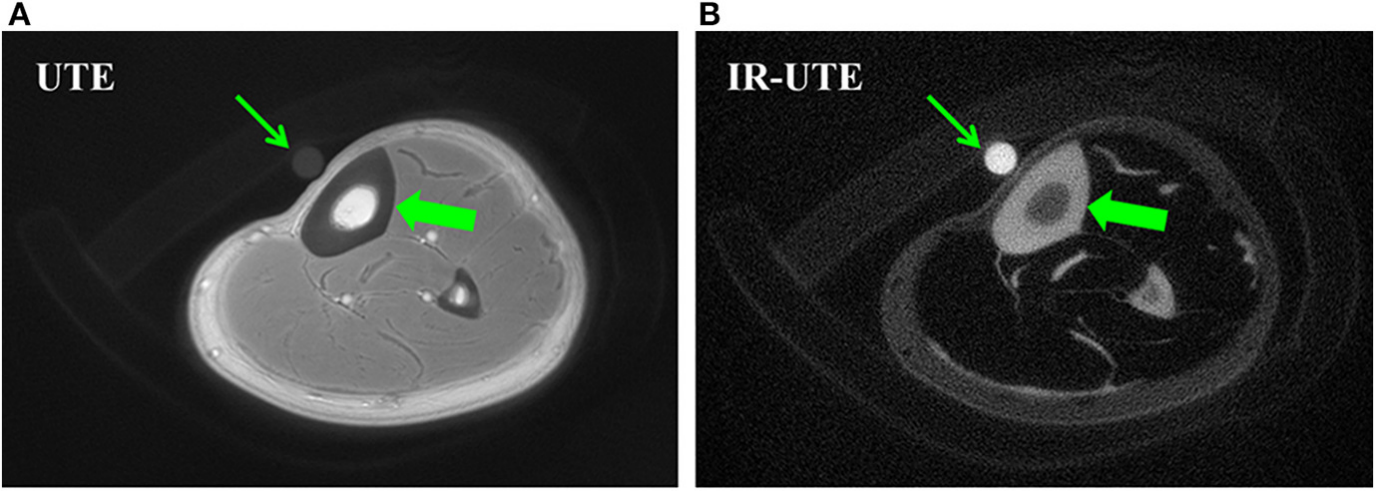
Bone water concentration was estimated by comparison of signal intensity of bone (thick arrows) relative to that of a water calibration phantom (thin arrows) using UTE **(A)** and IR-UTE **(B)** sequences, providing a bone water concentration estimation of 22.2 ± 2.7% and 16.8 ± 1.9%, respectively. This figure was previously presented by Du et al. (38). Reprinting permission is granted through Rightslink system. This figure is modified for presentation purposes. Minor modifications were performed for presentation purposes.

Figure 1 shows 2D UTE and 2D IR-UTE imaging of the tibial midshaft in a healthy young volunteer (38). A TW content of 22.2 ± 2.7% was found with basic UTE, and a BW content of 16.8 ± 1.9% was found with IR-UTE. A mixture of distilled water (20%) and D2O (80%) doped with 22 mM MnCl2 with similar T1 and T2^*^ values was used as the calibration phantom for TW measurement (38).

**Figure 2:**
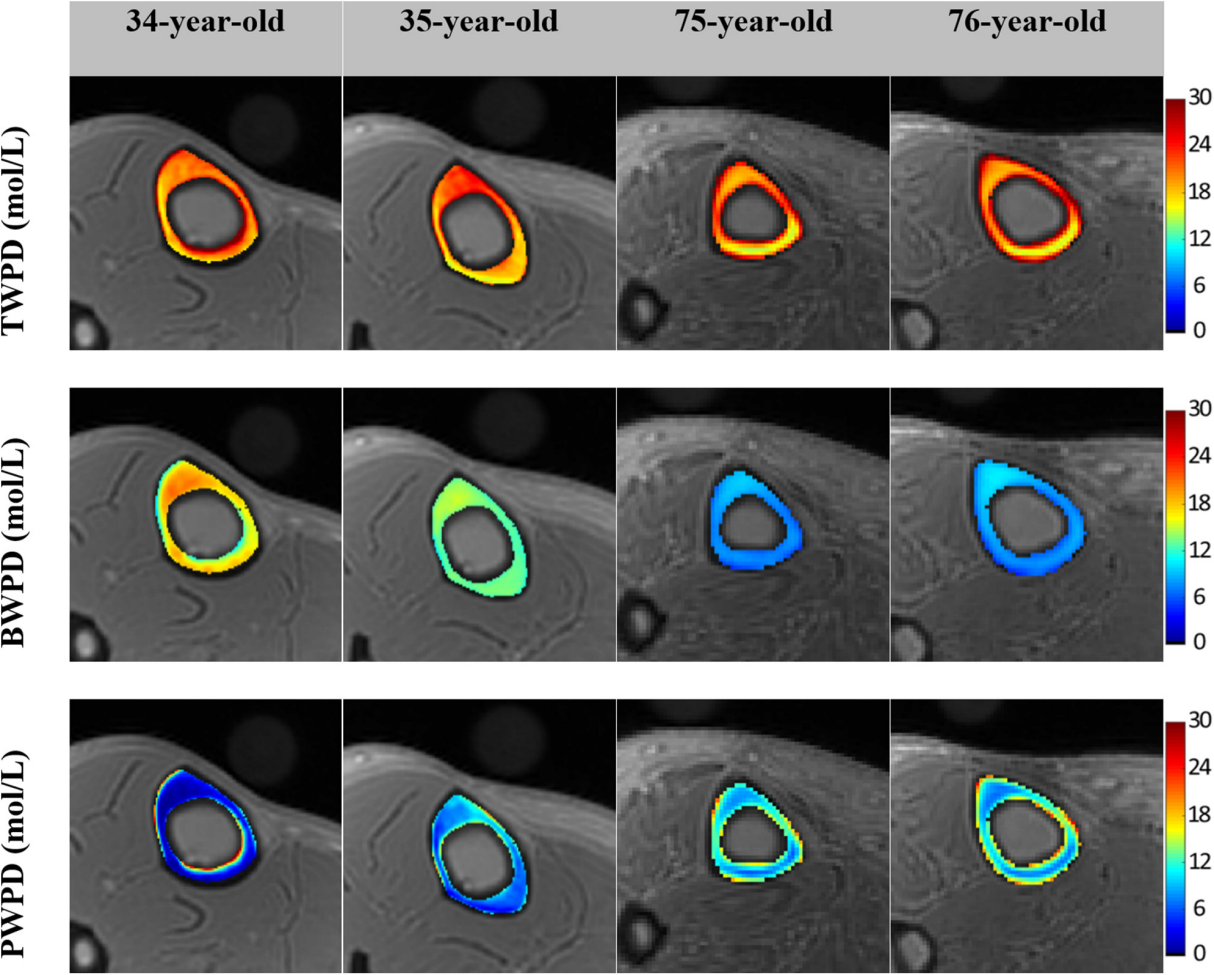
Generated TW proton density (TWPD), BW proton density (BWPD) and PW proton density (PWPD) maps for two young healthy volunteers (34- and 35-year-old female) and two old volunteers, (75- and 76-year-old female). In older individuals, PWPDs were higher and BWPDs were lower compared with the younger group. This figure was previously presented by Jerban et al. (44). Reprinting permission is granted through Rightslink system. This figure is modified for presentation purposes. Minor modifications were performed for presentation purposes.

Figure 2 shows *in vivo* TW proton density (TWPD), BW proton density (BWPD) and PW proton density (PWPD) maps for two young healthy and two old female volunteers (44). Qualitatively, PWPD was higher in older individuals compared with younger individuals. *Ex vivo* studies performed on human tibial cortex specimens have shown significant correlations between PWPD and the microstructural properties of bone (44).

BW and PW have also been estimated using dual-TR UTE imaging technique through a model-based UTE signal decomposition (57). PW content has been reported to correlate significantly with subject age (57).

### Double Adiabatic Full Passage Pulse (DAFP) UTE for Imaging of PW in Cortical Bone

**Figure 3:**
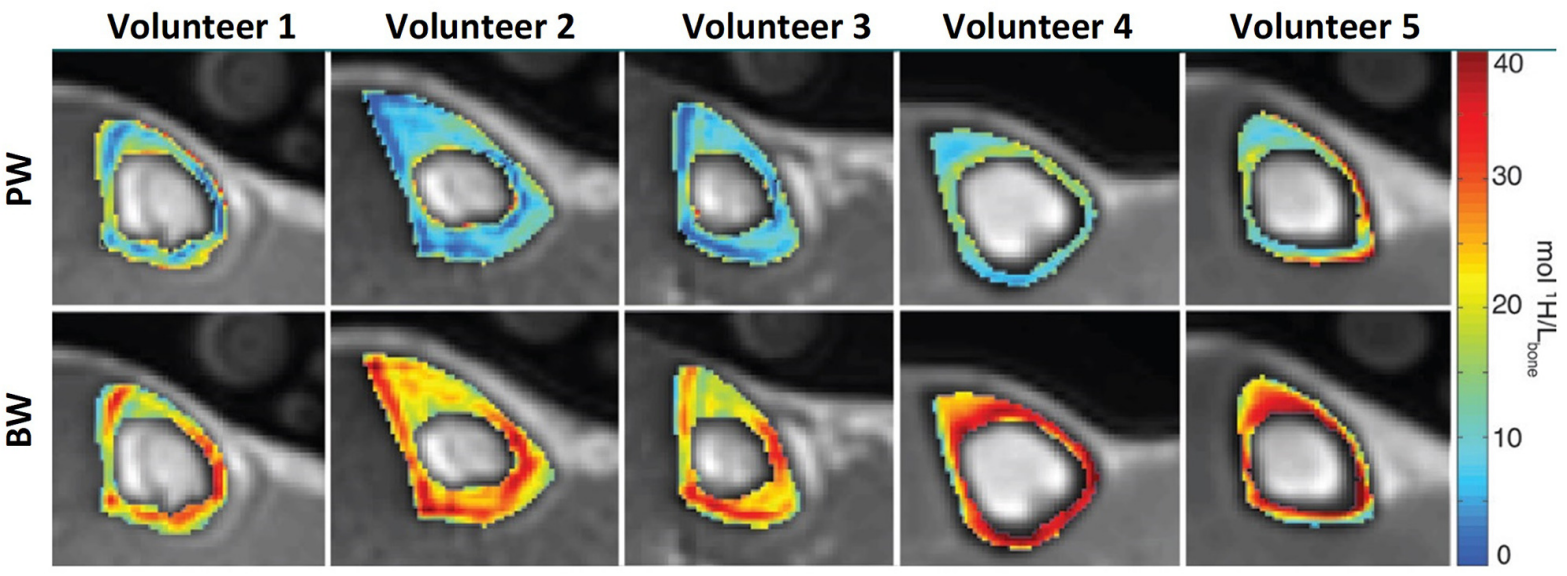
PW (top row) and BW (bottom row) proton density maps of tibial midshaft in five different subjects (two men and three women aged 24, 24, 49, 30, and 26 years). Maps are overlaid on UTE MRI images. This figure was previously presented by Manhard et al. (41). Reprinting permission is granted from Radiology journal (RSNA). This figure is modified for presentation purposes. Minor modifications were performed for presentation purposes.

Double adiabatic full passage pulse (DAFP) can directly image PW in cortical bone using a preparation to saturate the BW signal followed by a UTE acquisition (41, 47). DAFP technique requires an excellent nulling process of BW signal, which can be challenging to perform *in vivo*. Thus, indirect PW content estimation is likely more appealing compared with the direct approach using DAFP technique. Horch et al. (47) used UTE MRI at 4.7T for direct imaging of both BW and PW, and reported significant correlations with mechanical properties of bone strips. Later, Manhard et al. (48) demonstrated significant correlation between BW measured at 3T with the bone fracture toughness of cortical bone specimens. Figure 3 shows PWPD and BWPD in tibial cortical bone generated *in vivo* using direct PW (DAFP) and BW (IR-UTE or AIR-UTE) imaging (41).

The estimation of absolute water content using basic UTE, IR-UTE, and DAFP has the potential for translation to clinical cross-sectional and longitudinal investigations.

### Multi-Component UTE MRI Analysis in Cortical Bone

T2^*^ of PW is roughly ten times the T2^*^ of PW, and can be distinguished from one another using UTE MRI acquisition techniques combined with multi-component T2^*^ analysis (16, 58, 74). Such techniques, however, do not estimate absolute water proton contents, which makes them more appropriate for longitudinal studies. It should be noted that multi-component T2^*^ fitting at high strength magnetic fields may not be as reliable as is reported for lower field strengths (50). Multi-component T2^*^ fitting requires a series of MRI images with different TEs, which can extend the scanning process.

Bicomponent exponential T2^*^ fitting has been used in many studies to quantify BW and PW (12, 16, 51). Bae et al. (16) and Seifert et al. (50) found that BW and PW fractions obtained from bicomponent T2^*^ analysis were significantly correlated with human cortical bone porosity measured using μCT. Bae et al. also reported significant correlations between bicomponent T2^*^ results and mechanical properties of human cortical bone strips (16). Recently, the efficacy of UTE MRI bicomponent T2^*^ analysis was investigated by comparing with histomorphometric measures of bone porosity (51); Bicomponent T2^*^ was found to be capable of detecting bone porosities comprised of pores below the range detectable by μCT (51).

**Figure 4:**
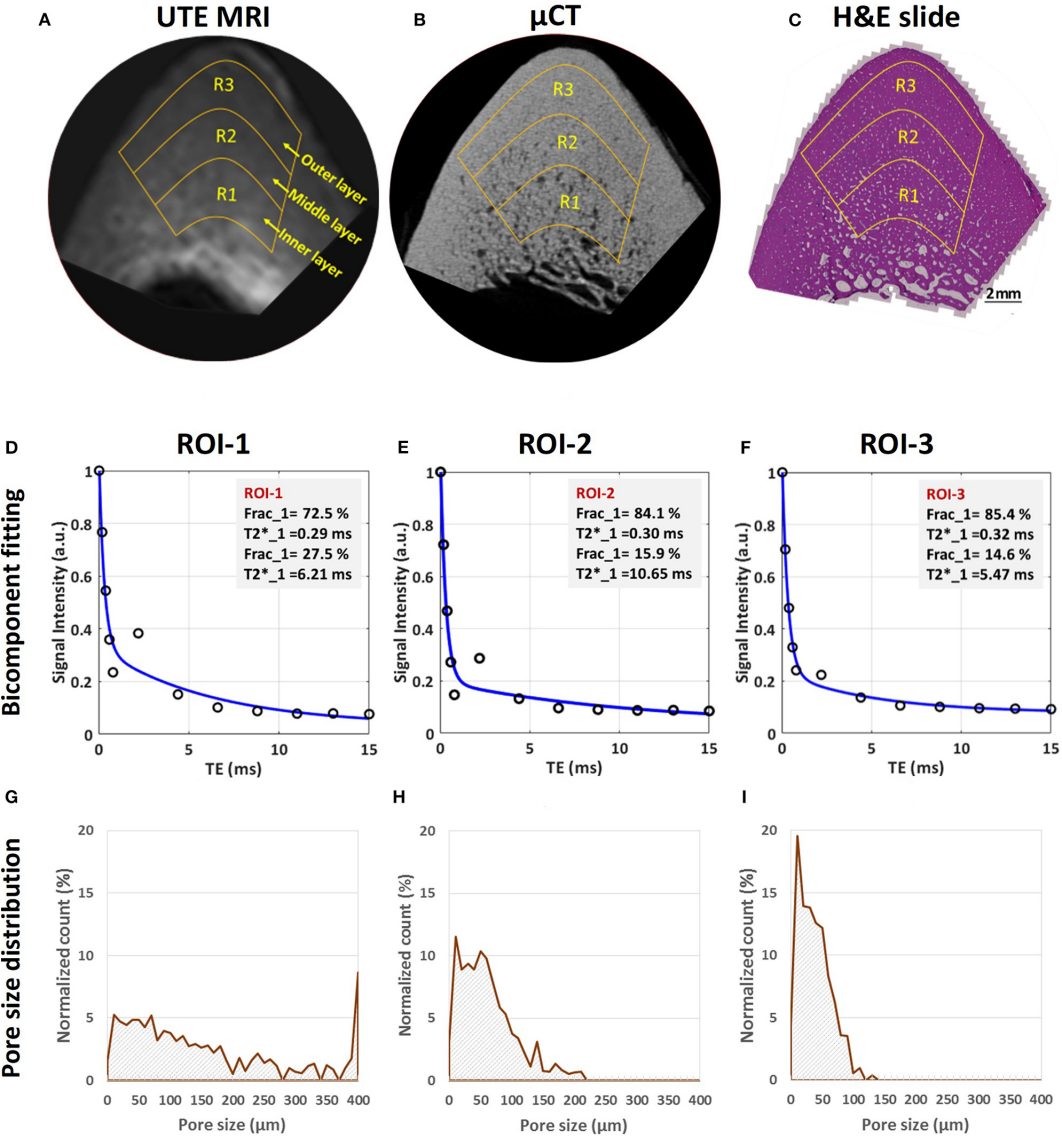
MRI-based and histomorphometric analyses for three representative ROIs at three different cortical bone layers. Selected ROIs at three different bone layers on a representative bone specimen (male, 71-year-old) illustrated on **(A)** UTE MRI (TE = 32 μs, 250 μm pixel size), **(B)** μCT (9 μm pixel size) and **(C)** histology (H&E-stained, 0.2 μm pixel size) images. Bicomponent exponential fitting of the T2* decay within **(D)** ROI-1, **(E)** ROI-2, and **(F)** ROI-3. The oscillating actual data points indicate the presence of fat particularly in ROI-1 and ROI-2 near the endosteum. Pore size distribution obtained from histomorphometric analyses are shown for **(G)** ROI-1, **(H)** ROI-2, and **(I)** ROI-3. Histomorphometric porosity and pore size for ROI-1 to−3 are 33.1, 13.9 and 7.1% and 221, 83 and 49 μm, respectively. The μCT-based porosities are 21.2, 8.2, and 1.7% for ROIs-1 to−3, respectively. This figure was previously presented by Jerban et al. (51). Reprinting permission is granted through Rightslink system. This figure is modified for presentation purposes. Minor modifications were performed for presentation purposes.

UTE MRI, μCT and histology images of a representative bone specimen (71-year-old male) are shown in Figure 4 (51). Bone layers closer to endosteum show higher porosity and larger pore size. Bicomponent T2^*^ fittings and the histomorphometry pore size distributions within the three bone layers are depicted in the second and third row subfigures. Short T2 fraction (Frac1) was found to be higher in regions with lower porosity and lower pore size (51). Peaks in pore size distributions shifted toward lower values for layers closer to the periosteum, indicating a limited number of large pores in the outer layers of cortical bone.

UTE bicomponent analysis was also utilized to study the effect of field strength on the T2^*^ of cortical bone at 1.5T and 3T (52). The BW T2^*^ and PW T2^*^ of human cortical bone were 21 and 68% lower, respectively, at 3T compared with 1.5T (52). However, BW and PW fractions showed only minor changes with field strength (<4%), suggesting that UTE bicomponent analysis may provide consistent BW and PW fractions at 1.5T and 3T, thereby allowing field-independent comparisons. Seifert et al. (50) later studied the performance of bicomponent analysis at higher magnetic fields (7T and 9.4T) and suggested that bicomponent analysis may fail at high magnetic fields, likely due to inaccurate fitting results cause by the difference between the short component T2^*^ and long component T2^*^ decreasing significantly at higher magnetic fields.

Human cortical bone possesses a considerable amount of fat, particularly in the regions near bone marrow. Average signal oscillation of the multi-echo MRI in T2 fitting analyses has been observed by different studies (11, 50, 54), a phenomenon explained most likely by the fat chemical shift (75). In order to remove fat signal contamination in bone water assessment, fat suppression techniques such as chemical shift fat saturation (FatSat), soft-hard water excitation and single point Dixon methods have been proposed (76, 77). FatSat is widely used in clinical MR sequences; however, it is not suitable for bone imaging due to the strong signal saturation of the wide spectrum band of bone. The novel soft-hard pulse has been proposed to overcome the signal attenuation effect by utilizing a low power soft-pulse for fat excitation in the opposite direction of the following hard pulse (76). Single-point Dixon method is a postprocessing method to separate water and fat signals for further analysis (77).

Tricomponent fitting model has been proposed to consider a modeled fat NMR spectrum (54), enabling improved estimation of BW and PW fractions in cortical bone. Estimation of water fraction by tricomponent T2^*^ fitting has improved correlation with μCT-based porosity compared to bicomponent fitting (53, 54). Tricomponent analysis has also shown higher correlation with the mechanical properties of bone (53). The tricomponent model avoids BW overestimation in the endosteal side of the cortex, a common error in bicomponent analysis (53, 54). However, the tricomponent model needs more data points which in turn requires a longer scan time, posing a challenge for translation to clinical applications.

**Figure 5:**
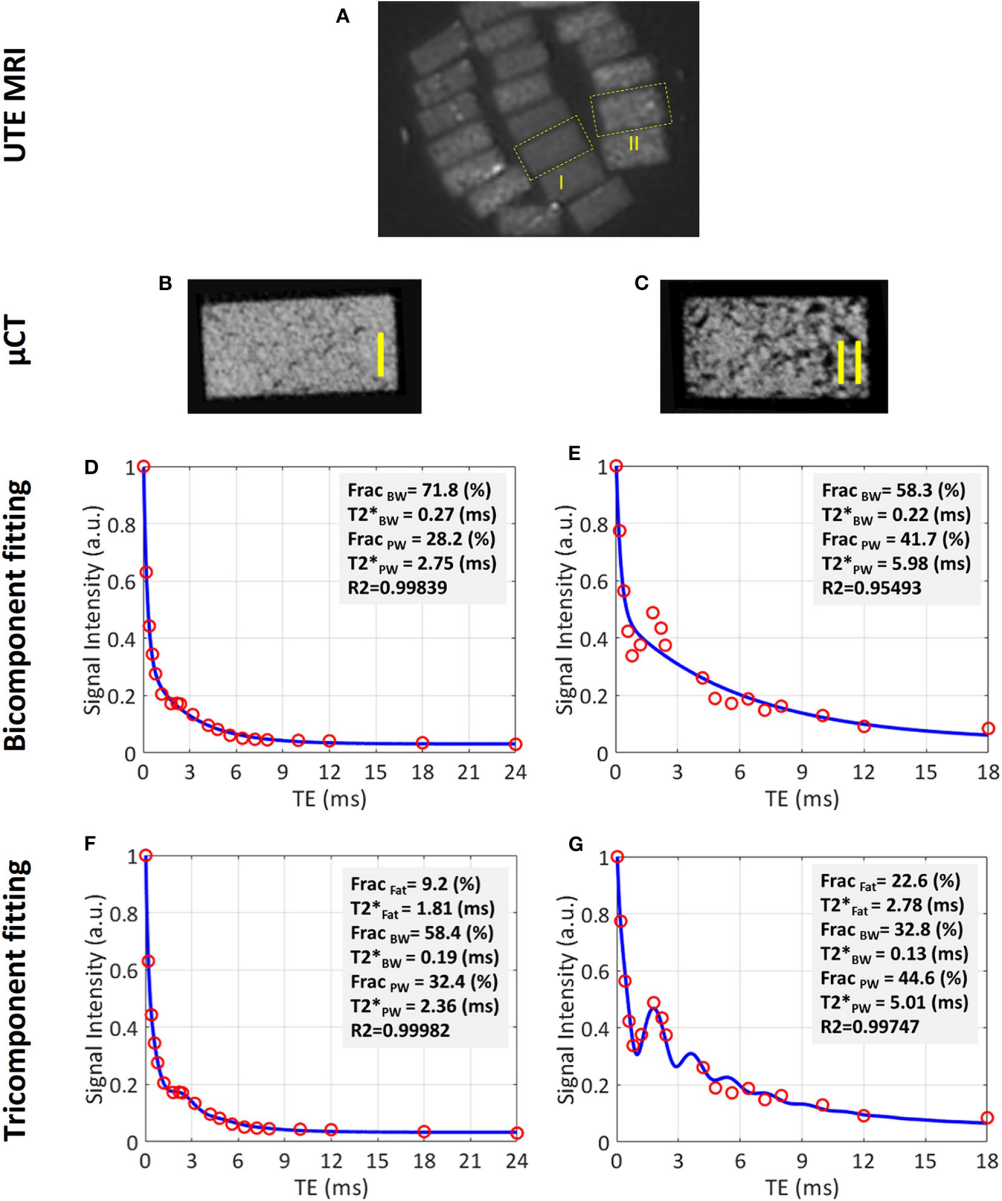
UTE MRI image and μCT images of two representative cortical bone strips harvested from different donors possessing different levels of porosities, in addition to bicomponent and tricomponent T2* fitting results. **(A)** UTE MRI (TE = 0.032 ms) image of a set of cortical bone strips with ~4 × 2 mm cross-sections soaked in fomblin, which has no signal in MRI. **(B,C)** μCT images of representative cortical bone strips from a 47-year-old male and 57-year-old female, respectively. **(D,E)** Bicomponent T2* fittings for the bone strips shown in **(B)** and **(C)**, respectively. **(F,G)** Tricomponent T2* fitting for bone strips shown in **(A,B)**, respectively. The oscillating signal decay in cortical bone specimens is better fitted by including the signal contribution of fat using the tri-component model (higher fitting R2 values). This figure was previously presented by Jerban et al. (53). Reprinting permission is granted through Rightslink system. This figure is modified for presentation purposes. Minor modifications were performed for presentation purposes.

Figure 5A shows a UTE MRI image covering a set of bone specimens with 4 × 2 mm cross-sections placed in a 1-inch birdcage coil. Figures 5B,C illustrate the μCT images of samples I and II with 15% and 33% average porosities, respectively (53). Bicomponent and tricomponent fitting analyses are demonstrated in Figures 5D–G for both specimens. Sample II shows a large oscillating signal which has been well-fitted using the tricomponent model.

### UTE MRI Fractional Indexes in Cortical Bone

Dual echo time UTE imaging (55) can be used to calculate the so-called porosity index (PI), which is the signal ratio between two MRI images, one with TE ≈ 0.05 ms and one with TE ≈ 2 ms. The first echo image represents signal from both BW and PW, and the second echo represents mostly PW signal. Although this technique does not estimate the absolute PW content, it gives an estimation of bone porosity. PI in human cadaveric tibiae has shown significant correlations with μCT-based porosity, mechanical stiffness, donor age and collagen estimation from near infrared spectroscopy (55, 56). This technique is much faster that the multi-component fitting analyses even though the obtained ration between PW to TW is likely more accurate when calculated with multi-component techniques.

Suppression ratio (SR), defined as the ratio between bone UTE signal without long T2 suppression and with long T2 suppression performed via dual-band saturation-prepared UTE (DB-UTE) or IR-UTE, is another UTE MRI-based index that has been proposed for evaluation of cortical bone microstructure (40). SR can be considered as the TW to BW ratio in cortical bone. This technique requires faster MR imaging compared with multi-component fitting techniques. It should be noted that Bone from older subjects showed higher SR values (40). Similarly, *ex vivo* investigations have shown that SR demonstrates significant correlations with bone porosity and donor age (40). This technique is much faster than the multi-component fitting analyses even though the obtained ration between PW to TW is likely more accurate when calculated with multi-component techniques. PI and SR ratios do not provide absolute estimations of bone water contents like multi-component analyses do, making them more appropriate for longitudinal studies.

### UTE Magnetization Transfer (UTE-MT) Imaging of Cortical Bone

Direct quantification of collagen backbone protons is very challenging with current MRI scanners because the collagen protons possess extremely short T2^*^ (59). Magnetization transfer (MT) imaging combined with UTE MRI is suggested to indirectly assess protons in the collagenous matrix (60, 61). With MT techniques, a high-power saturation RF pulse is applied with a frequency offset from the water resonance frequency to saturate the magnetization of collagen protons. The saturated magnetization can transfer from the collagen to water protons, which can then be imaged with UTE MRI. UTE-MT assessment of collagen protons, such as MTR, has been shown to be significantly correlated with bone microstructural and mechanical properties (62).

The magnitude of the transferred saturation is a function of the macromolecular proton fraction (MMF). MMF, as well as macromolecular proton relaxation time (T2_mm_) and exchange rates, can be obtained using two-pool modeling performed on UTE-MT data acquired with a series of RF pulse powers and frequency offsets (60). MMF from UTE-MT modeling has shown strong correlation with both human bone microstructure measured via μCT and histomorphometry (51, 61) and with mechanical properties (44, 51, 58, 61). Although UTE-MT modeling requires a relatively longer MRI scan time compared with basic UTE and IR-UTE methods for TW and BW content estimations, respectively, it provides a unique quantification of the collagenous matrix of bone. The MMF estimation is more appealing if a bone disease affects the collagenous matrix independently from the bone volume and BMD, such as is the case in osteomalacia disease (78, 79).

**Figure 6:**
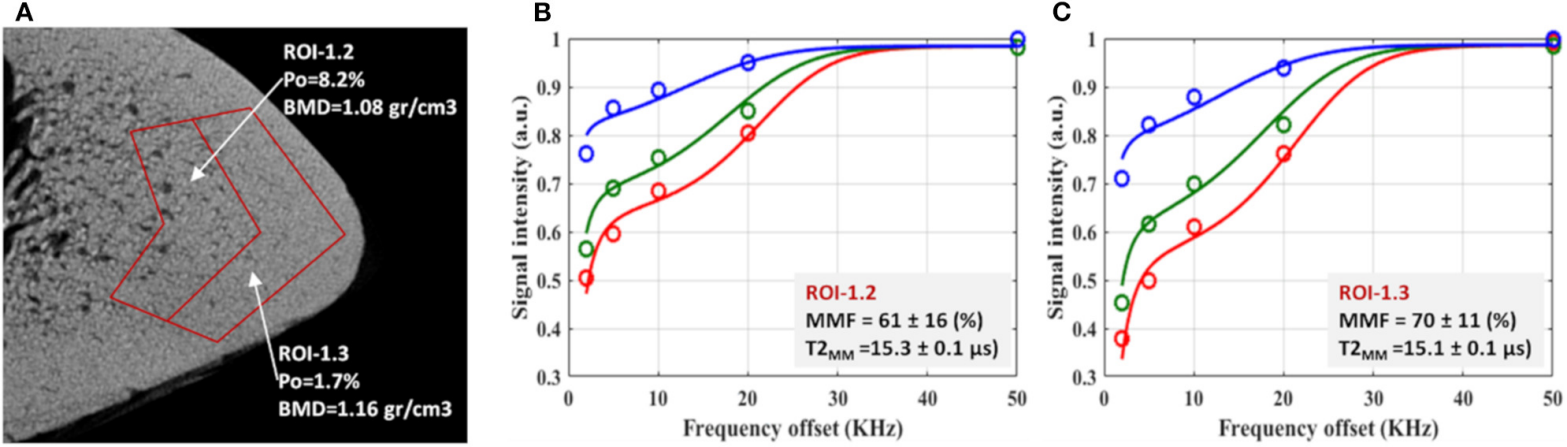
**(A)** μCT image of a representative tibial specimen (male, 73-year-old) focused on anterior tibia with two selected ROIs in middle and outer layers. Measured porosity (Po) in middle layer (ROI-1.2) is higher than that of outer layer (ROI-1.3). The two-pool MT modeling analyses in **(B)** ROI-1.2 and **(C)** ROI-1.3 using three pulse saturation powers (500° in blue, 1,000° in green and 1,500° in red) and five frequency offsets (2, 5, 10, 20, 50 kHz). MMF and T2_MM_ refer to macromolecular fraction and macromolecular T2, respectively. This figure was previously presented by Jerban et al. (61). Reprinting permission is granted through Rightslink system. This figure is modified for presentation purposes. Minor modifications were performed for presentation purposes.

Figure 6 shows the relationship between bone microstructure and UTE-MT modeling results (61). Figure 6A illustrates a zoomed μCT image of a representative tibial bone specimen focused on the anterior tibia. Porosity and BMD are measured for two selected regions in the middle and outer layers of the cortex. Two-pool MT modeling analyses of the selected regions of interest (ROIs) are shown in Figures 6B,C, respectively, using three MT saturation pulse powers (500°, 1,000°, and 1,500°) and five off-resonance frequencies (2, 5, 10, 20, and 50 kHz).

**Figure 7:**
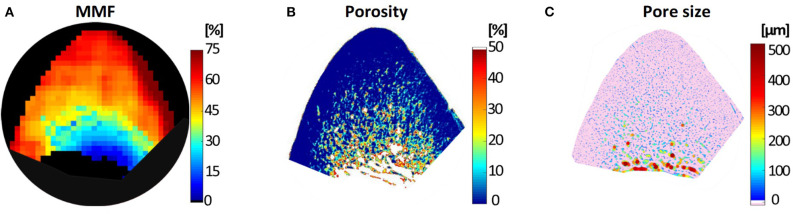
**(A)** Macromolecular fraction (MMF) from MT modeling, **(B)** μCT-based porosity and **(C)** histomorphometry-based pore size maps of a representative anterior tibial bone specimen (male, 71-year-old). This figure was previously presented by Jerban et al. (51). Reprinting permission is granted through Rightslink system. This figure is modified for presentation purposes. Minor modifications were performed for presentation purposes.

Generated MMF, μCT-based porosity and histology-based pore size maps for a similar representative specimen are shown in Figure 7 (51). The MMF pixel map demonstrates an increasing pattern toward outer bone layer, where both μCT and histology indicate a low porosity.

**Figure 8:**
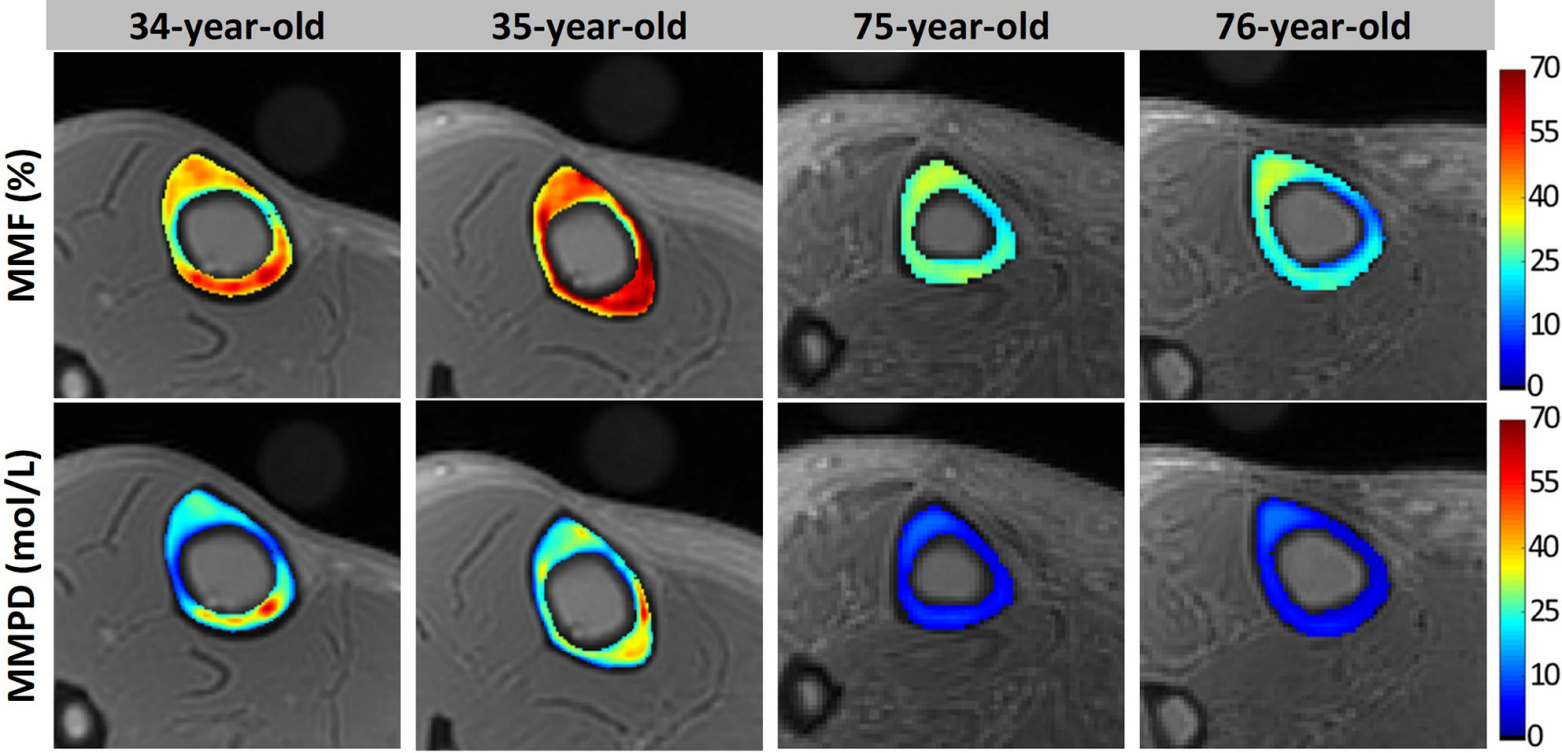
Generated MMF and macromolecular proton density (MMPD) maps for two young healthy volunteers (34- and 35-year-old females) and two old volunteers (75- and 76-year-old females). In older individuals, MMF and MMPD were lower compared with the younger group. This figure was previously presented by Jerban et al. (44). Reprinting permission is granted through Rightslink system. This figure is modified for presentation purposes. Minor modifications were performed for presentation purposes.

Macromolecular proton density (MMPD) can be calculated as a function of MMF and TWPD (44). Figure 8 shows *in vivo* MMF and MMPD maps for two young healthy and two old female volunteers. MMF and MMPD appeared higher in younger individuals compared with the elderly group (44). MMPD measure can be considered superior to MMF because it predicts the absolute content of the macromolecules in bone which can be used in both cross-sectional and longitudinal investigations.

### UTE Quantitative Susceptibility Mapping (UTE-QSM) Assessment of Bone Minerals

**Figure 9:**
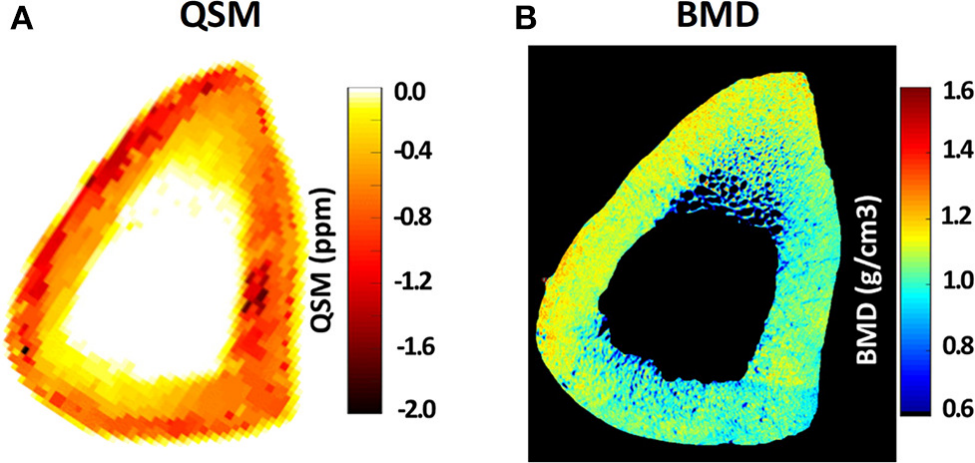
**(A)** Quantitative susceptibility map (QSM) using Cones 3D UTE MRI scans (0.5 × 0.5 × 2 mm voxel size) of a representative tibial midshaft cortical bone (45-year-old female), **(B)** μCT-based volumetric bone mineral density (BMD) map of the same specimen. Local maxima in the QSM map clearly correspond to the regions of high BMD in μCT-based maps. This figure was previously presented by Jerban et al. (64). Reprinting permission is granted through Rightslink system. This figure is modified for presentation purposes. Minor modifications were performed for presentation purposes.

Quantitative susceptibility mapping (QSM) de-convolves magnetic susceptibility of the tissue based on the phase changes in the MR signal, such that tissues with stronger magnetic susceptibility undergo faster evolution of phase. Dimov et al. (63) developed the UTE-QSM technique for potential detection of mineral variations in porcine hoof and human distal femur. They reported significant correlations between radial 3D UTE-QSM values and computed tomography (CT) Hounsfield units in a combined set of ROIs covering tendon, trabecular bone, and cortical bone. Recently, UTE-QSM has been investigated in human tibial cortical bone specimens, and significant correlations between QSM and BMD have been reported (64). Figure 9 illustrates Cones UTE-QSM and volumetric BMD maps for a representative cortical bone specimen from tibial midshaft. Local maxima of the QSM map qualitatively correspond to the regions of high BMD in μCT-based maps (64). UTE-QSM technique requires a much longer scan time than basic UTE technique. UTE-QSM paired with UTE-MT, and basic UTE are capable of multi-component bone evaluations.

### UTE ^31^P Imaging for Assessment of Bone Minerals

**Figure 10:**
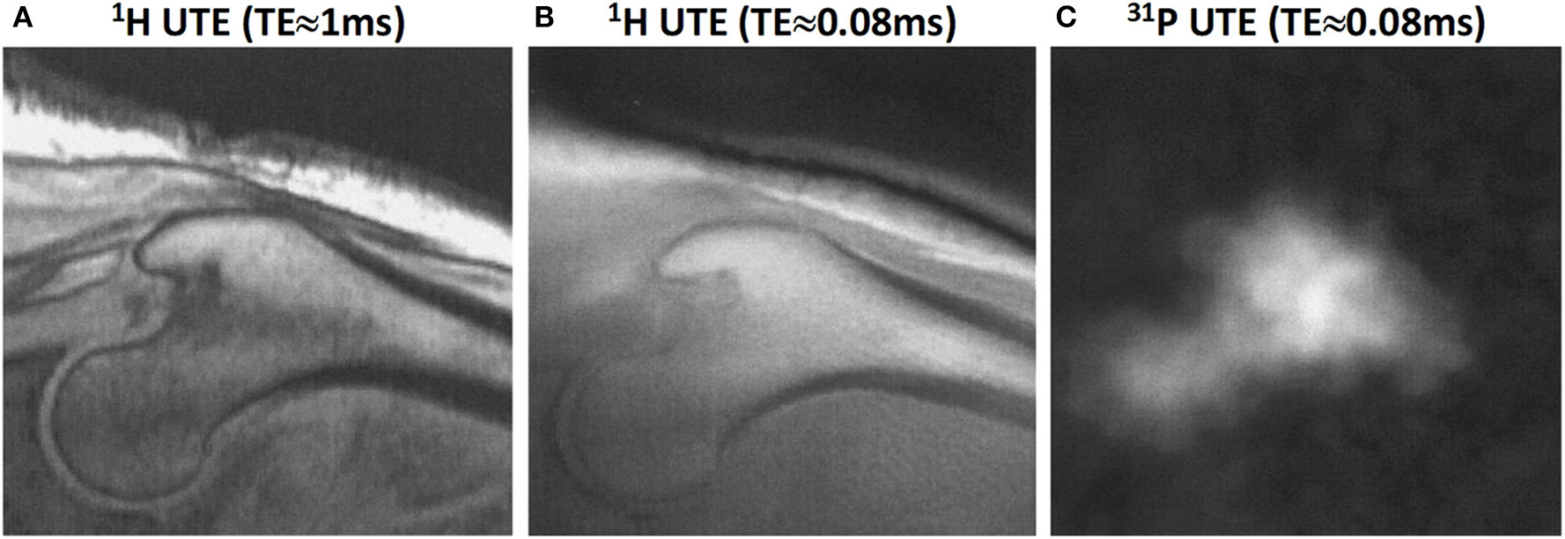
Proximal femur bone images in a male subject (58-year-old). **(A)** ^1^H proton image at TE≈1 ms, **(B)** ^1^H proton UTE image at TE≈0.08 ms, and **(C)** ^31^P UTE image at TE≈0.08 ms. This figure was previously presented by Robson et al. (66). The reprinting permission is granted through Rightslink system. This figure is modified for presentation purposes. Minor modifications were performed for presentation purposes.

Phosphorus (i.e., ^31^P) imaging combined with UTE, water- and fat-suppressed proton projection (WASPI) or zero echo time (ZTE) MR acquisitions have been employed for bone mineral estimation in several studies (42, 43, 65). The feasibility of *in vivo* ^31^P imaging in human subjects has been shown at 1.5T UTE-based tibia and femoral head imaging (66). Figure 10 shows the femoral head of middle-aged subject imaged using ^1^H and ^31^P UTE imaging (66). Phosphorus quantification has the potential to differentiate between mature calcified bone and newly remodeled bone. However, translating the phosphorus imaging to clinical investigations will be challenging as the required instruments are not available in most scanners.

## UTE MRI Quantification of Trabecular Bone

High resolution MR imaging of bone marrow using clinical sequences has been suggested for indirect visualization of trabecular bone as dark regions surrounded by marrow with a high signal intensity. The 3D microstructural parameters of trabecular bone can be obtained following few image post-processing steps (6, 80, 81). Both gradient-echo and spin-echo clinical acquisitions have been reported for high resolution trabecular bone assessment (82). To the authors' knowledge, this approach has not been reported in the literature using basic UTE MRI because of the high resolution and long scan time requirements. Moreover, UTE MRI results in lower image contrast between bone and soft tissue compared with clinical sequences, which challenges the post-processing steps. High magnetic susceptibility in trabecular bone sites is an additional barrier for employing basic UTE via this approach.

Direct trabecular bone imaging is technically challenging because of the fast signal decay of bone as implied by its short T2 (25). To create a high contrast for trabecular bone in proton imaging, it is critical to suppress signals from long T2 tissues, particularly the marrow fat. Wurnig et al. (67) used the UTE sequence to visualize trabecular bone *ex vivo* and to measure their T2^*^ values at different magnetic fields. This direct trabecular bone imaging was achieved through a SPIR (spectral pre-saturation with inversion recovery) module to suppress marrow fat. Investigating T2^*^ values in trabecular bone regions showed significant correlations with bone microstructural parameters obtained from μCT (67). However, innate sensitivity of these techniques to B1 and B0 inhomogeneities may limit the clinical applications of these techniques.

**Figure 11:**
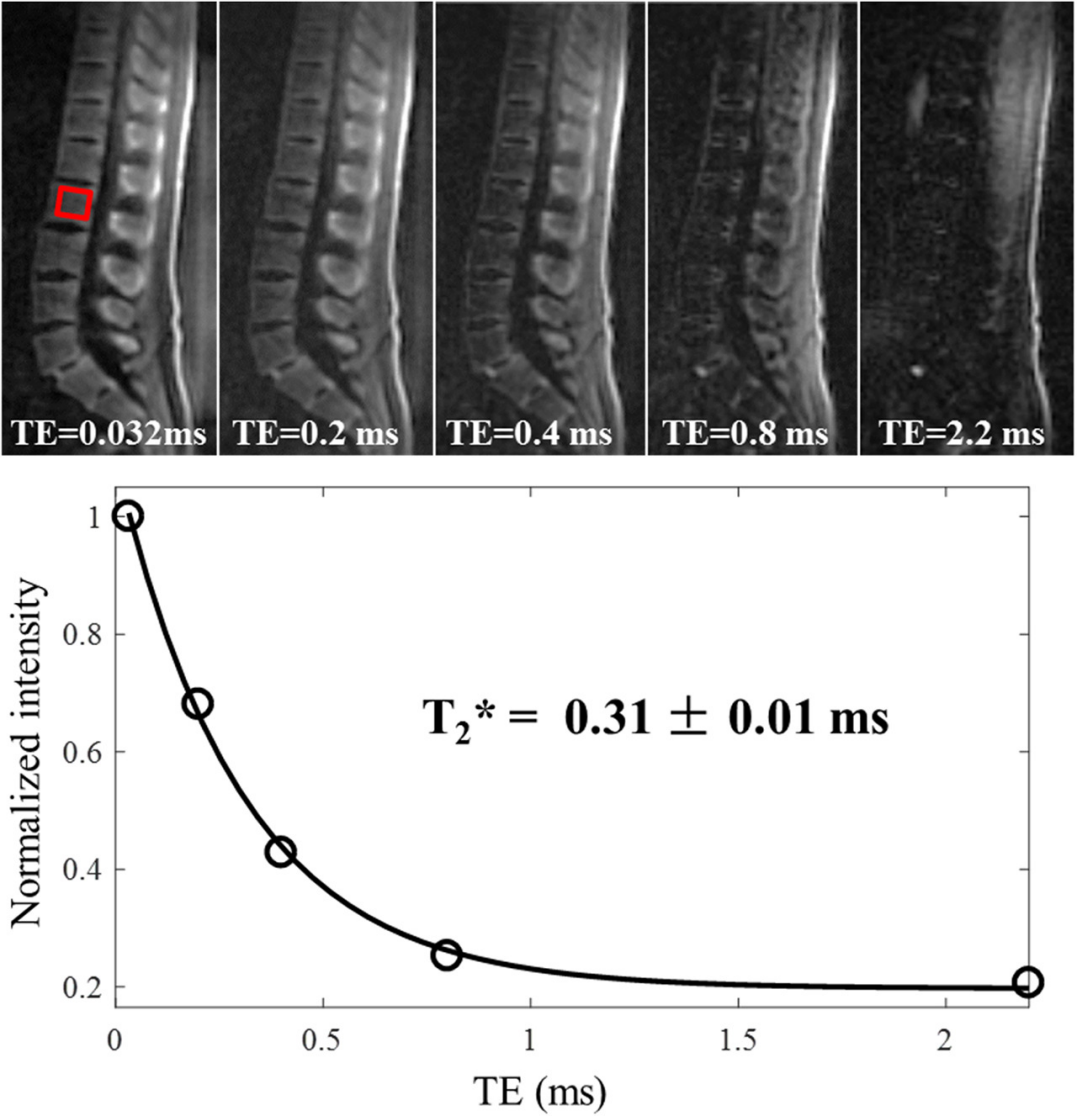
*In vivo* imaging of the spine of a 36-year-old male volunteer using the 3D IR-UTE-Cones sequence with TEs of 0.032, 0.2, 0.4, 0.8, and 2.2 ms. Single-component fitting is achieved for a selected vertebra with a short T2* of 0.31 ± 0.01 ms, which demonstrates that long T2 water and marrow fat are sufficiently suppressed in the IR-UTE-Cones images. This figure was previously presented by Ma et al. (68). Reprinting permission is granted through Rightslink system. This figure is modified for presentation purposes. Minor modifications were performed for presentation purposes.

3D adiabatic IR-UTE Cones (3D IR-UTE-Cones) sequence has been proposed by Ma et al. (68) to directly visualize trabecular bone and measure relaxation times (68). A broadband adiabatic inversion pulse was used together with a short TR/TI combination to suppress signals from long T2 tissues such as muscle and marrow fat. The suppression is followed by multi-spoke UTE acquisition to detect signal from short T2 water components in trabecular bone. This technique provides low sensitivity to B1 and B0 inhomogeneities due to the use of broadband adiabatic inversion pulses (68). The developed techniques have been applied *ex vivo* and *in vivo* at 3T and resulted in valid ranges of T2^*^ values (0.3–0.45 ms) and proton densities (5–9 mol/L) for trabecular bone. *In vivo* 3D IR-UTE-Cones images of the lumbar spine at different TEs (0.032 to 2.2 ms) are shown in Figure 11, in addition to the corresponding T2^*^ curve fitting. The fitted T2^*^ is very close to that of cortical bone, suggesting efficient suppression of signals from bone marrow fat.

**Figure 12:**
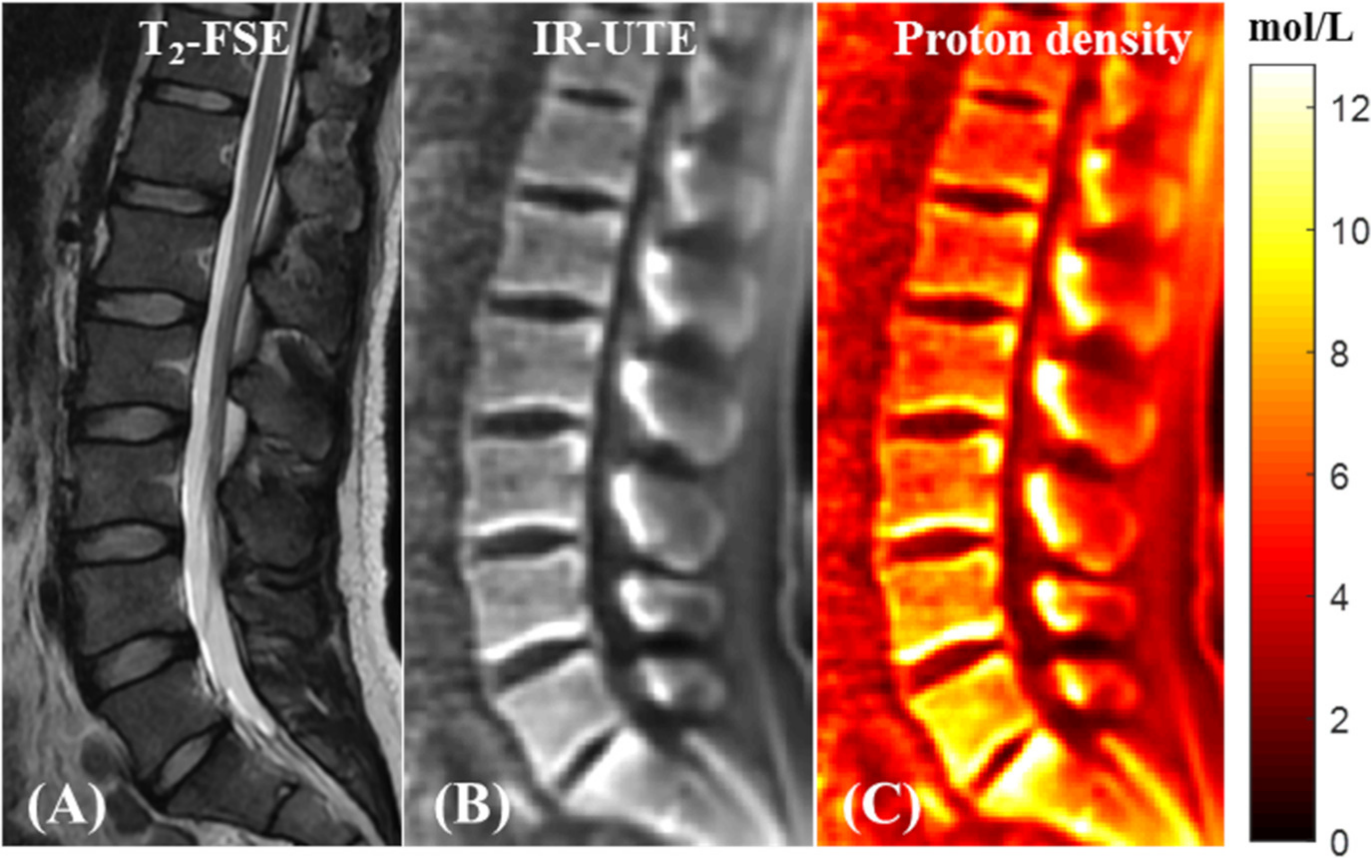
*In vivo* qualitative and quantitative imaging of the spine of a 31-year-old male volunteer using the 3D IR-UTE-Cones sequence. The long T2 muscle and fat are bright in the clinical T2-FSE image **(A)**. **(B)** 3D IR-UTE-Cones image after coil sensitivity correction. **(C)** PD map of the spine trabecular bone. This figure was previously presented by Ma et al. (68). Reprinting permission is granted through Rightslink system. This figure is modified for presentation purposes. Minor modifications were performed for presentation purposes.

Bound water proton density mapping can be achieved for trabecular bone by comparing its signal obtained from 3D IR-UTE-Cones imaging and that of an external reference phantom with a known proton density (68). Figure 12 shows the T2-weighted FSE and IR-UTE images of a healthy volunteer in addition to the water proton density map in the lumbar spine (68).

## Conclusions

Quantitative UTE MRI assessment of different water, collagen and mineral compartments of both cortical bone and trabecular bone have been of great interest to orthopedic research society. Several quantitative MR techniques are discussed for assessment of cortical and trabecular bone. UTE techniques enable TW quantification in cortical bone using clinical whole-body scanners. IR-UTE-based techniques provide BW assessment. Long T2-saturated UTE sequences, such as WASPI, can also provide selective imaging of BW *ex vivo* and *in vivo*. UTE multi-component T2^*^ analysis can distinguish between BW and PW T2^*^ and their fractions. UTE-MT can potentially provide information of collagen content in cortical bone. Other UTE type techniques, such as ZTE, DAFP and WASPI have the potential to provide quantitative measurements of bone water compartments.

## Author Contributions

Y-JM, SJ, and JD contributed to the experimental design. Y-JM and SJ contributed to data collection. All authors contributed to data analysis, writing, and interpretation.

## Conflict of Interest

The authors declare that the research was conducted in the absence of any commercial or financial relationships that could be construed as a potential conflict of interest.
